# Simulated sample heating from a nanofocused X-ray beam

**DOI:** 10.1107/S1600577517008712

**Published:** 2017-08-02

**Authors:** Harald Wallander, Jesper Wallentin

**Affiliations:** a Synchrotron Radiation Research, Lund University, Box 118, Lund 22100, Sweden

**Keywords:** heating, radiation damage, simulation, nanostructures

## Abstract

Recent developments in synchrotron brilliance and X-ray optics are pushing the flux density in nanofocusing experiments to unprecedented levels, which increases the risk of different types of radiation damage. The effect of X-ray induced sample heating has been investigated using time-resolved and steady-state three-dimensional finite-element modelling of representative nanostructures. Simulations of a semiconductor nanowire indicate that the heat generated by X-ray absorption is efficiently transported within the nanowire, and that the temperature becomes homogeneous after about 5 ns. The most important channel for heat loss is conduction to the substrate, where the heat transfer coefficient and the interfacial area are limiting the heat transport. While convective heat transfer to air is significant, the thermal radiation is negligible. The steady-state average temperature in the nanowire is 8 K above room temperature at the reference parameters. In the absence of heat transfer to the substrate, the temperature increase at the same flux reaches 55 K in air and far beyond the melting temperature in vacuum. Reducing the size of the X-ray focus at constant flux only increases the maximum temperature marginally. These results suggest that the key strategy for reducing the X-ray induced heating is to improve the heat transfer to the surrounding.

## Abstract 2

Time-resolved finite-element modelling is used to study the sample heating from intense X-ray irradiation.

## 1. Introduction   

In the field of crystallography of biological macromolecules, radiation damage has been a problem and a research topic for decades (Garman & Weik, 2017 ▸). Radiation damage leads to loss of resolution, and is believed to be induced by free radicals, bond breaking and heating (Coughlan *et al.*, 2017 ▸). Hard condensed matter is generally much less sensitive to radiation damage, although recent reports indicate, for instance, X-ray induced reduction of metal ions (Stanley *et al.*, 2014 ▸). Driven by the need for higher spatial resolution, an increasing number of synchrotron beamlines with nanofocusing capabilities have recently become available (Tamasaku *et al.*, 2001 ▸; Riekel *et al.*, 2010 ▸; Schroer *et al.*, 2010 ▸; Winarski *et al.*, 2012 ▸; Johansson *et al.*, 2013 ▸; de Jonge *et al.*, 2014 ▸; Nazaretski *et al.*, 2015 ▸; Salditt *et al.*, 2015 ▸; Somogyi *et al.*, 2015 ▸; Martínez-Criado *et al.*, 2016 ▸). The next years will see further enhancements in both flux and focusing. Typical nanofocus sizes now reach ∼100 nm, with demonstrations of techniques for sub-10 nm focusing (Mimura *et al.*, 2010 ▸; Döring *et al.*, 2013 ▸), while the introduction of diffraction-limited storage rings will radically improve the coherent flux (Eriksson *et al.*, 2014 ▸). Combined, these improvements will enhance the flux densities by several orders of magnitude.

The power density absorbed by the samples will inevitably increase as well, which increases the risk of radiation damage in condensed matter samples. Here, we consider one aspect of radiation damage, heating, which can lead to permanent damage, such as structural changes or oxidation, but also non-destructively affect measured results through, for instance, thermal expansion, sample movement, increased chemical activity or electrical conductance. The sensitivity to heating varies widely between experiments, but in, for example, low-temperature physics or live cell experiments even an increase of a few degrees Kelvin could affect results. We present simulations of sample heating in nanostructures exposed to nanofocused X-rays. The system that we studied in greatest detail is an InP nanowire deposited on a Si_3_N_4_ membrane (Wilke *et al.*, 2014 ▸), but we also present brief results for a few other systems such as gold nanoparticles.

Synchrotrons are pulsed X-ray sources, with a typical pulse length of 0.1 ns and period of a few nanoseconds. During the experiment, some of the X-ray photons are absorbed in the sample due to photoelectric absorption. After the primary photoelectric absorption, the core hole and the photoelectron can generate secondary photons and electrons. Some of these secondary particles can leave the sample and carry away energy, in processes that depend strongly on X-ray energy, photoelectron escape depth, sample composition and geometry, but a large part of the energy is eventually converted into heat. We assume that all of the absorbed X-ray power is instantaneously converted into heat, which is an overestimate, in order to focus on the heat transport.

After absorption and conversion to heat, the energy is dispersed through different processes. The heat is distributed to colder regions within the sample, which in the time-dependent case is described by the heat equation,
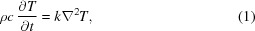
with the density ρ, the specific heat capacity per unit mass *c*, the thermal conductivity *k*, and the time and spatially dependent temperature *T*. The heat is also transported to the surroundings through heat transfer and radiation. The heat flux across an interface, *q*, can be described by 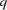 = 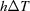, where *h* is the heat transfer coefficient, which is also known as the gap conductance, and Δ*T* is the temperature difference at the interface (not the Laplace operator). At the interface to air, *h* is the convective heat transfer coefficient. Finally, thermal radiation should be considered, described by 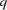 = 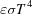, where ∊ is the emissivity and σ is the Stefan–Boltzmann constant.

Together, these processes form a complex time-dependent three-dimensional heat transfer problem. We have investigated this problem using finite-element modelling, which is an established method for studying heating in X-ray optics (Hoszowska *et al.*, 2001 ▸). First, we present time-dependent simulations of the single nanowire under the reference conditions. Thereafter, we use steady-state simulations to vary several of the parameters in the model. Finally, we show steady-state simulations of a few other nanostructures.

## 2. Method   

**Figure 1:**
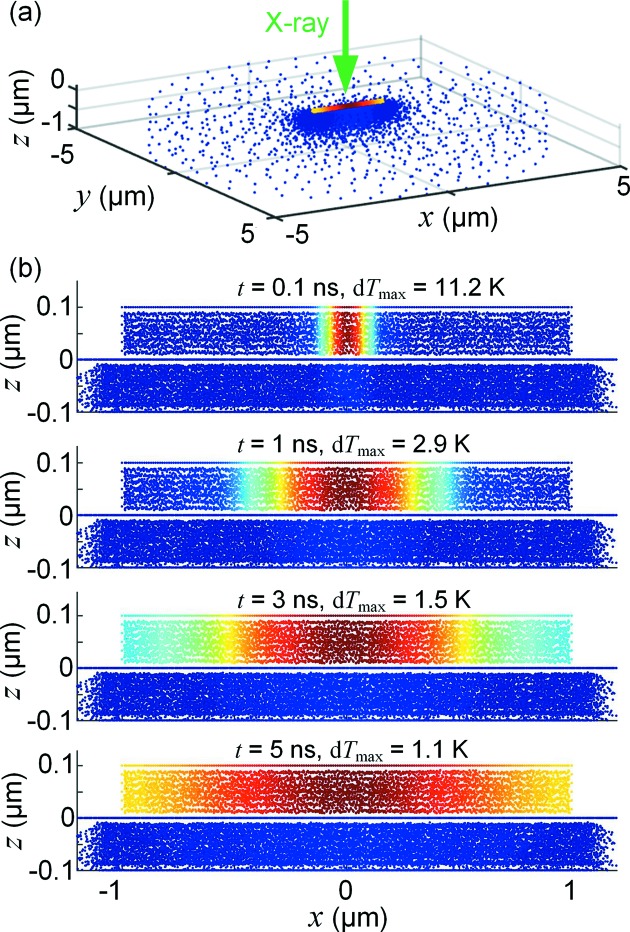
(*a*) Overview of the simulated experiment, showing an InP nanowire on a Si_3_N_4_ membrane. Each node in the finite-element mesh is indicated with a dot. (*b*) Simulated temperature relative to room temperature, d*T*, at different times after absorption of a single X-ray pulse of length 0.1 ns, as seen along the *y*-axis. Note that the colour scale is different for each time. The full simulation can be found as Video 1 in the supporting information.

The geometry of the sample was drawn using the software *COMSOL Multiphysics* (version 5.2, COMSOL AB, Stockholm, Sweden), and relevant materials parameters were defined. The 2 µm-long nanowire was modelled with a hexagonal cross section, with a diameter of *d* = 100 nm between two flat facets. The Si_3_N_4_ substrate was modelled as a circular slab of thickness 1 µm and diameter 5 µm. The temperature at the circumference of the slab was fixed at room temperature, and the other interfaces were surrounded by air. The finite-element mesh was defined to be denser in the nanowire and in the section of the substrate near the nanowire, with about 10 nm distance between nodes (Fig. 1*a* ▸). Radiative heat exchange between the nanowire and the substrate was not accounted for in the simulations, since it was computationally demanding but physically insignificant except at unreasonably high temperatures. Different mesh densities were compared to verify that the results were independent of the mesh. A regular personal computer was used for calculations, with typical simulation times that ranged from minutes to hours.

In general, the beam has a non-uniform intensity profile and the sample has a non-uniform thickness and attenuation length, which means that the absorbed power must be calculated for each point in the sample. The X-ray beam was modelled with a Gaussian intensity profile with a full width at half-maximum (FWHM) diameter of *D* = 100 nm. The beam was incident in the negative *z*-direction, with the nanowire aligned along the *x*-axis (Fig. 1*a* ▸). As a reference case, a photon flux of Φ = 10^12^ photons s^−1^ was used (energy 10 keV, power *P*_0_ = 1.6 mW), which is near the upper end of reported fluxes from current beamlines. In the case of hard X-rays and thin samples such as nanostructures, the attenuation length is much longer than the sample thickness. The Beer–Lambert law can then safely be linearized, such that the absorbed intensity *P*_abs_ in a sample of thickness *z* is 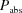 = 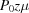, where *P*_0_ is the intensity of the primary beam and μ is the absorption coefficient. Since the absorbed power is proportional to both the flux and the absorption coefficient, an increased absorption coefficient, due to for instance a lower X-ray energy or higher atomic mass in the sample, is equivalent to an increased flux.

The program calculates the absorbed power at each point using the absorption coefficient, which was calculated from materials data assuming a 10 keV X-ray photon energy. For InP, the material which was used for most of the nano­structures, μ_InP_ = 0.0505 µm^−1^. Below a flux of about 10^11^ photons s^−1^, the average number of absorbed photons per pulse is below 1. This means that the deposited energy is a stochastic process, where a single 10 keV absorption event deposits energy in a much smaller region than the focus size. However, we did not consider such effects.

Tabulated emissivity data were used to model the radiative cooling, and we assumed a room temperature of 20°C. Bulk data for thermal conductivity was used, *k* = 68 W K^−1^ m^−1^ for InP, although recent reports show that it can be reduced by about an order of magnitude in nanostructures due to phonon scattering at the surface (Lee *et al.*, 2016 ▸). The reported heat transfer coefficients across solid–solid interfaces, *h*, vary widely in the literature, not just between different material combinations. Heat is only transferred in the small regions of atomic contact, and the fraction of such a contact area varies by several orders of magnitude depending on surface roughness and hardness (Ohsone *et al.*, 1999 ▸). Interfaces between evaporated metal and insulators have recently been reported to have heat transfer coefficients of around 30–200 MW m^−2^ K^−1^ (Cahill *et al.*, 2003 ▸; Siemens *et al.*, 2010 ▸; Oyake *et al.*, 2015 ▸). Since the metal is free to form an almost perfect interface in an evaporation process, these values can be seen as an upper limit for a solid–solid interface. For the evaporated Au–InP interface in the contacted nanowire, as well as the Au–Si interface in the Au droplet, we used a heat transfer coefficient of 100 MW m^−2^ K^−1^. In our case, we assumed that crystalline InP nanowires were mechanically deposited from their growth substrate onto a Si_3_N_4_ membrane. Nanowires normally have quite smooth surfaces, but still show roughness and facets on the nanometer scale (Hjort *et al.*, 2013 ▸). We therefore used a value of 10 MW m^−2^ K^−1^ for the nanowire–substrate interface.

For the last sample, a model bacterium, we assumed that the density was 1.35 g cm^−3^ and the composition H_50_C_30_N_9_O_10_S (Howells *et al.*, 2009 ▸). The thermal conductivity was set at *k* = 0.6 W K^−1^ m^−1^ (Kyoo Park *et al.*, 2013 ▸), slightly lower than water, while the heat transfer coefficient to the silicon substrate was approximated with a water–silicon interface, *h* = 100 MW m^−2^ K^−1^ (Ramos-Alvarado *et al.*, 2016 ▸).

## 3. Time-resolved simulations   

**Figure 2:**
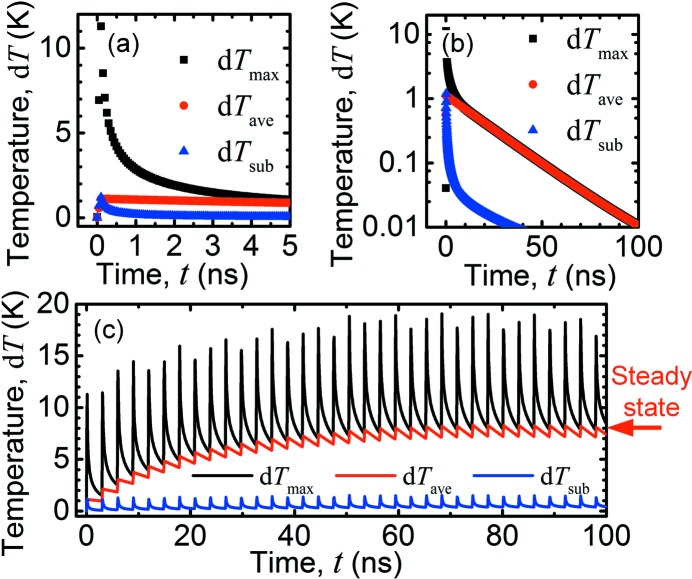
Time dependence of the temperatures: maximum and average temperature in the nanowire, d*T*_max_ and d*T*_ave_, respectively, and the maximum temperature in the substrate, d*T*_sub_. (*a*) Single pulse, the first 5 ns, linear plot. (*b*) Single pulse, the first 100 ns, semi-logarithmic plot. The decay time constant of d*T*_ave_ is 20.8 ns. (*c*) Repeated pulses with 3 ns period, linear plot. The arrow indicates d*T*_ave_ from the steady-state simulations. The full simulation can be found as Video 2 in the supporting information.

First, we studied the time-dependent response from single and repeated pulsed X-rays (pulse period 3 ns). The time-averaged flux of the repeated pulses was Φ = 10^12^ photons s^−1^, *i.e.* 3000 photons per pulse, and we used the same number of photons for the single pulse. Fig. 1(*b*) ▸ shows the temperature distribution within the sample at different times after a single pulse of length 0.1 ns, starting at *t* = 0. In Fig. 2 ▸, we have plotted the time dependences of the maximum (d*T*_max_) and average (d*T*_ave_) temperature in the nanowire, and the maximum temperature in the substrate (d*T*_sub_), defined relative to room temperature. In the plot with repeated pulses, an arrow indicates the temperature from a steady-state simulation, where we used an X-ray beam with the same average power as the pulsed beam (see next section[Sec sec4]). The full time evolution with the single pulse and with the repeated pulses can be found as Video 1 and 2, respectively, in the supporting information.

The single-pulse response shows that, immediately after the pulse has been absorbed, d*T*_max_ = 11.3 K and d*T*_ave_ = 1.1 K. The heat spreads quickly within the nanowire, for instance d*T*_max_ (*t* = 3 ns) = 1.5 K, and after about 5 ns the temperature is almost homogeneous. The decay of d*T*_ave_ is slower, showing an exponential decay with a time constant of 20.8 ns (Fig. 2*b* ▸). The time scales are similar to calculations of nanostructures heated by pulsed lasers (Sassaroli *et al.*, 2009 ▸; Chen *et al.*, 2012 ▸). For the repeated pulses with 3 ns pulse period, the time is insufficient for d*T*_ave_ to fully decay to room temperature between pulses (Fig. 2*c* ▸). Instead, d*T*_ave_ gradually increases to a stable level around d*T*_ave_ = 8 K. After reaching the stable level, d*T*_ave_ and d*T*_max_ oscillate with amplitudes of 1.0 K and 11 K, respectively, in agreement with the single-pulse simulation.

Although the absorption and heat conduction is a complicated three-dimensional problem, we can gain some insight with simplified analytical models. We note that the absorbed power in the nanowire is of the order of 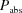 ≃ 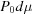 ≃ 8 µW, which is a slight overestimate since the nanowire is less than 100 nm optically thick for much of its cross section. If we consider that the energy per pulse is *E*_pulse_ = 30 MeV = 4.8 pJ, the absorbed energy per pulse is approximately 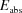 ≃ 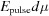 ≃ 24 fJ = 150 keV, *i.e.* 15 photons. Ignoring heat dissipation, which should be a valid assumption at very short time scales, the temperature increase in a volume *V* is given by d*T* = 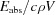. Assuming that all of the energy is absorbed in a 100 nm-long segment of the nanowire, we find d*T* ≃ 19 K. Similarly, we can estimate the increase of d*T*_ave_ using the full 2 µm length to calculate *V*, and find d*T* ≃ 0.9 K. Indeed, these two values are similar to the amplitudes of the short-time oscillations of d*T*_max_ and d*T*_ave_.

We can also model the temporal dependence with analytical arguments, by assuming that the cooling after the X-ray pulse proceeds in two independent processes: heat distribution within the nanowire and heat transfer to the surrounding. Although these actually proceed simultaneously, we can treat them separately for simplicity. Considering the first process, we can write the heat equation in one dimension,
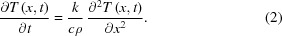
This partial differential equation lacks a simple analytical solution, but the characteristic time scale of the problem is given by 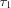 = 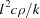, where *l* is the characteristic length (Langtangen & Pedersen, 2016 ▸). For a point halfway between the focus and the end of the nanowire, *l* = 0.5 µm, we find τ_1_ ≃ 5 ns, which is in reasonable agreement with the full simulations. On the other hand, at the length scale of the focal radius, *l* = 50 nm, we find *τ*_1_ ≃ 50 ps, which is less than the pulse length. This means that, even for a very small focus, the energy will dissipate within about 100 nm at the time scale of the X-ray pulse length. However, note that we assumed that the X-ray energy is instantaneously transferred to the lattice, while for instance bandgap recombination can take about 1 ns even in direct semiconductors. An accurate description of X-ray heating at time scales below 1 ns probably requires a different methodology, which treats the X-ray absorption as discrete events at points in space.

In the second cooling process, as will be shown more clearly in the steady-state simulations below, the energy dissipates mainly by heat transfer to the substrate. Assuming that the nanowire can be described by a single homogeneous time-dependent temperature *T*(*t*), and a constant substrate temperature *T*_sub_, the heat flux to the substrate is 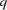 = 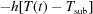. The heat flux and temperature are related *via* the heat capacity as 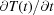 = 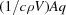, where *A* is the area of one facet and *V* is the volume. The temperature is then described by the equation
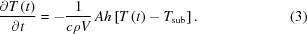
With 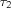 = 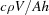, we can write the solution as 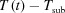 = 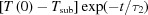, which is an exponential decay. Using our reference values, we find the time constant τ_2_ ≃ 26 ns, in good agreement with the full simulation. Thus, the analytical models and the full simulations show that the temperature decays in two processes with different time constants τ_1_ and τ_2_. We can also make some predictions about how the time constants depend on the sample parameters, as will be investigated in the next section. While τ_1_ is inversely proportional to *k*, τ_2_ is inversely proportional to *h*. As *V* ≃ *d* ^2^ and *A* ≃ *d*, we expect τ_2_ to increase linearly with *d*, *i.e.* slower cooling to the substrate for larger diameters.

## 4. Steady-state simulations   

We are primarily interested in the sample temperatures at typical exposure times, which are much longer than the pulse period in most synchrotron experiments, because we want to study their dependence on geometry and material parameters. Since full time-resolved simulations are computationally demanding, we employed steady-state simulations in 3D for a wide range of parameters. In this case, we modelled the X-ray beam with constant flux at the same average flux as the pulsed beam. The steady-state simplification is justified by the relatively small short-term temperature oscillations, as discussed above. In order to validate this assumption, we also performed full time-resolved simulations at selected parameter values (not shown). For a significantly longer bunch distance, such as in single-bunch timing modes or free-electron lasers, the temperature oscillations will be much larger than the steady-state temperature increases, and the steady-state simplification becomes a poor model. However, the general trends of heat dissipation, regarding sample size and materials parameters, should still be valid. For clarity, we describe the three-dimensional temperature with three representative temperatures, namely the maximum (*T*_max_) and average (*T*_ave_) temperature in the nanowire, and the maximum temperature in the substrate (*T*_sub_).

**Figure 3:**
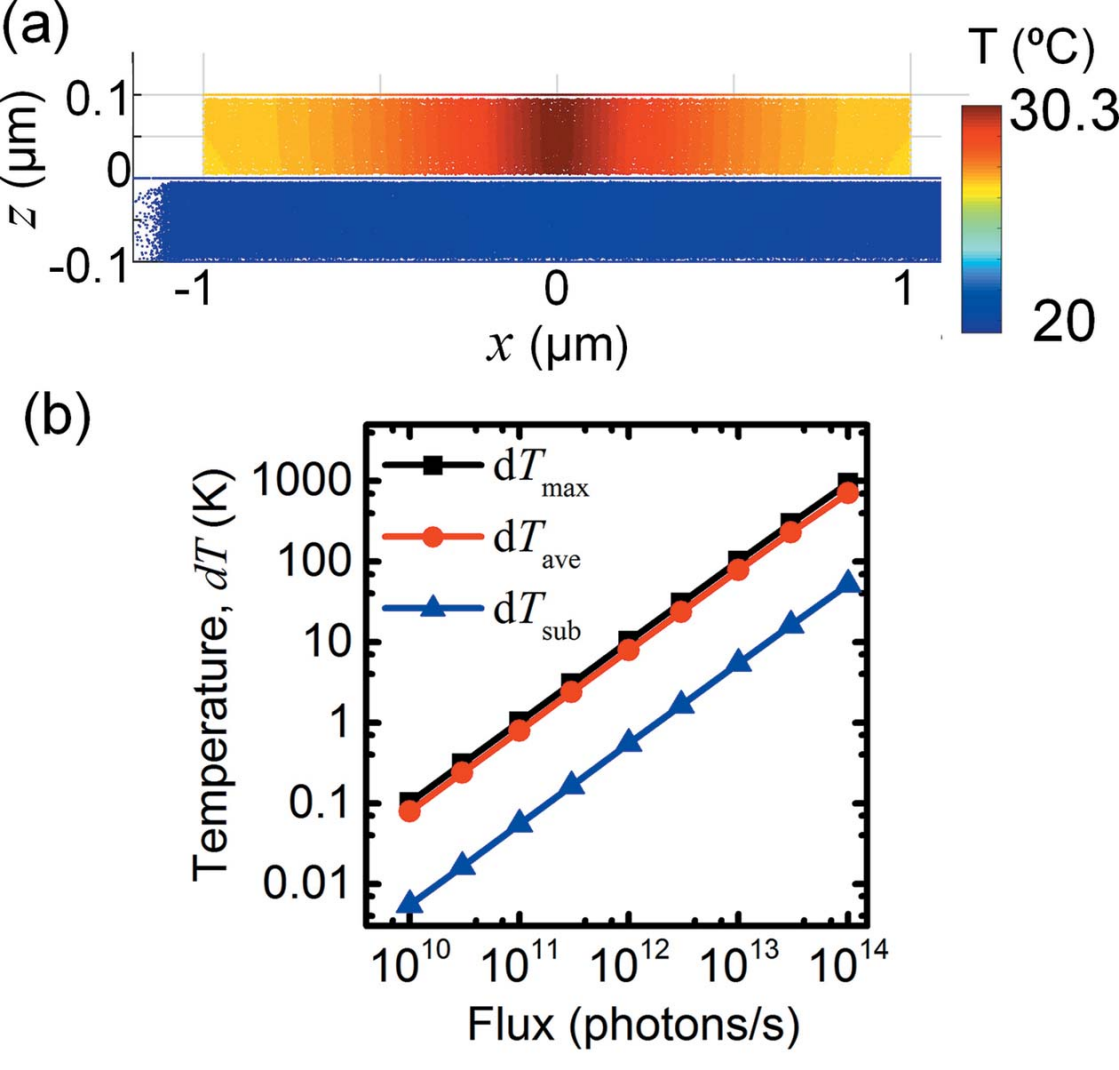
Steady-state three-dimensional simulations. (*a*) Image of the temperature in the sample, seen along the *y*-axis. (*b*) Simulated temperatures, relative to room temperature, *versus* X-ray flux.

The sample temperature at the reference parameters, projected along the *y*-axis, is shown in Fig. 3(*a*) ▸. We find that *T*_max_ = 30.3°C and *T*_ave_ = 28.0°C, while *T*_sub_ = 20.08°C. Thus, there is a relatively small temperature gradient within the nanowire, and a very small gradient within the substrate. The increase of the average temperature, d*T*_ave_ = 8 K, is in good agreement with the temperature at the end of the time-resolved simulation (Fig. 2*b* ▸).

The first parameter we studied was the X-ray flux, which was varied over several orders of magnitude with all other parameters fixed, as shown in Fig. 3(*b*) ▸. We find that all three temperatures are proportional to the flux. Throughout the entire range, the main temperature difference is between the nanowire and the substrate, not within the nanowire. At the highest flux, 10^14^ photons s^−1^, the steady state temperature is above 700°C. Time-dependent simulations at 10^10^ and 10^14^ photons s^−1^ display the same time constants as at 10^12^ photons s^−1^.

**Figure 4:**
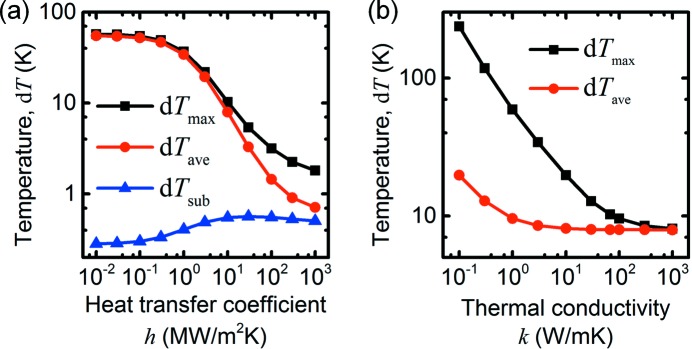
Simulated temperatures, relative to room temperature, *versus* (*a*) heat transfer coefficient, *h*, at the nanowire–substrate interface, (*b*) thermal conductivity, *k*, in the InP nanowire.

Next, we varied the heat transfer coefficient, *h*, and the heat conductivity, *k*, as shown in Fig. 4 ▸. There is a wide range of reported values for the heat transfer coefficient across solid–solid interfaces, as discussed in the *Method* [Sec sec2] section, and the simulations show that it has a strong influence on the steady-state temperature (Fig. 4*a* ▸). For *h* = 0, the simulated temperature reaches d*T*_ave_ = 55.1 K, about seven times more than at the baseline parameters. Time-dependent simulations at *h* = 1000 MW m^−2^ K^−1^ show the same *τ*_1_ but a very short *τ*_2_, as expected from the discussion in the previous section. Conversely, at *h* = 0.01 MW m^−2^ K^−1^, τ_1_ is also the same, while τ_2_ is much longer. Note that a low heat transfer coefficient is equivalent to a small interfacial area. We modelled a nanowire with hexagonal cross section, but more facets or even cylindrical cross sections are also common and in such cases the interfacial area can be drastically reduced. Obviously, a shorter nanowire would also have a smaller interfacial area and therefore higher temperature.

The nanowire dissipates heat *via* two other channels, aside from the heat transfer to the substrate: convective heat transfer to the surrounding air and thermal radiation. We therefore simulated the importance of these channels separately. At the reference heat transfer coefficient, *h* = 10 MW m^−2^ K^−1^, removing the convection only raised the average temperature from *T*_ave_ = 28.0°C to *T*_ave_ = 29.1°C. However, in the absence of heat transfer to the substrate, *h* = 0, additionally removing the convection increased the temperature from *T*_ave_ = 75.1°C to *T*_ave_ = 4250°C. This shows that thermal radiation is a very inefficient mode of heat transfer. Such experimental conditions could be reached, for instance, with a sample in vacuum with poor adhesion to its substrate.

Thus, we find that there is a clear hierarchy of the three cooling channels, with heat transfer to the substrate dominating under reference conditions. The heat is transferred efficiently within the solids, giving a relatively homogeneous temperature within the nanowire and within the substrate (*T*_sub_ ≃ *T*_room_). The cooling is limited by heat transfer from the nanowire to the substrate. At steady state, assuming an interfacial area *A*, the absorbed power *P*_abs_ must be balanced by heat transfer: 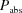 = 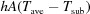. The nanowire temperature is therefore given by 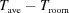 ≃ 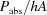. Since *P*_abs_ ≃ Φ, we find that *T*_ave_ ≃ Φ, as can be observed in Fig. 3(*b*) ▸. The slope, the thermal resistance *R* = d*T*/d*P*, is about 1.2 × 10^6^ K W^−1^, which is similar to results from modelling of a Si nanowire on a Si substrate (Bahadur *et al.*, 2005 ▸).

InP is a relatively good heat conductor, but the thermal conductivity *k* can vary by several orders of magnitude between different materials. To investigate the temperature in other materials, this parameter was varied in a range from *k* = 0.1 W m^−1 ^ K^−1^, corresponding to plastics, to *k* = 1000 W m^−1 ^ K^−1^, which corresponds to diamond (Fig. 4*b* ▸). The specific heat capacity, which varies much less, was left constant at the value of InP (*c* = 310 J kg^−1^ K^−1^). We find that at high thermal conductivity, as expected, the maximum temperature decreases to the average temperature, *T*_max_ ≃ *T*_ave_, and the internal temperature gradient disappears. *T*_ave_ remained essentially constant, however, since it is limited by the heat transfer coefficient. Time-resolved simulations at *k* = 1000 W m^−1 ^ K^−1^ showed that τ_1_ was very small, *i.e.* the temperature was immediately homogeneous within the nanowire, while τ_2_ was unaffected.

At lower thermal conductivity, the maximum temperature in the nanowire increases significantly, while the average one changes much less. At sufficiently low thermal conductivity, the average temperature is also limited by the thermal conductivity in the nanowire. Under these conditions, the central part of the nanowire is significantly warmer than the rest, and the steady-state cooling is limited by the heat transfer to the substrate with a much smaller interfacial area. Consequently, time-resolved simulations at *k* = 0.1 W m^−1 ^ K^−1^ showed that τ_1_ was much larger than at the reference value, while τ_2_ was slightly larger. In reality, a material with such low thermal conductivity, such as plastics, also has a low heat transfer coefficient.

**Figure 5:**
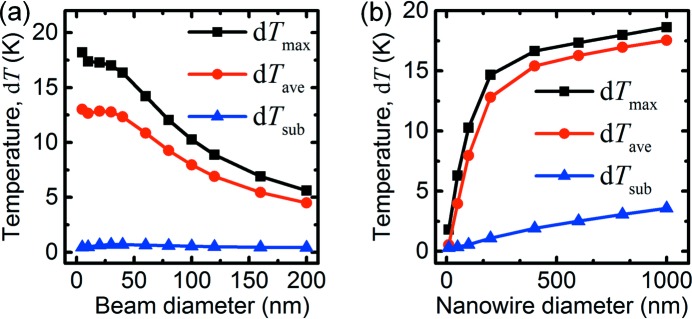
Simulated temperatures, relative to room temperature, *versus* (*a*) X-ray beam diameter, (*b*) nanowire diameter.

Next, we investigated the influence of the beam diameter, *D*, and the nanowire diameter, *d* (Fig. 5 ▸). Reducing the beam diameter from 100 nm to 10 nm increases *T*_max_ only by about 7 K and *T*_ave_ by about 5 K (Fig. 5*a* ▸). Time-resolved simulations showed that the oscillations of *T*_max_ were much larger at the smallest beam diameter, about 40 K compared with 11 K at the reference, although the validity of our assumptions at such short time and length scales is questionable. The decay time constants were approximately independent of beam diameter. The small steady-state temperature increase is due to increased X-ray absorption. At *D* = 100 nm, a significant part of the beam passes outside the thickest region of the nanowire, but, for *D* < 50 nm, the size of the nanowire facets, practically the entire beam passes through the thickest part of the nanowire. The small increase at *D* = 10 nm is a computational artefact, since the beam size is similar to the distance between nodes in the grid. Conversely, increasing the beam size above 100 nm reduces the overlap and therefore the temperature.

In a similar way, increasing the nanowire diameter increases the temperature through increased absorption (Fig. 5*b* ▸). For *d* > 200 nm, the overlap between the beam and the nanowire is essentially complete. The absorbing thickness is equal to the diameter, giving a linear increase of the absorbed power (*P*_abs_ ≃ *d*), but, since the interfacial area also increases as *A* ≃ *d*, the temperature is approximately constant. The small increase is due to substrate heating. Reducing the nanowire diameter below 100 nm reduces both the beam–nanowire overlap and the absorbing thickness, *i.e.* the absorbing volume decreases as ∼*d* ^2^, while the interfacial area decreases only as *A* ≃ *d*. Overall, this gives an approximately linear drop in average temperature. As expected, τ_2_ increases with the nanowire diameter. Note that we used bulk data for the thermal conductivity, although recent reports indicate strongly reduced thermal conductivity in nanostructures (Lee *et al.*, 2016 ▸).

## 5. Other sample geometries   

**Figure 6:**
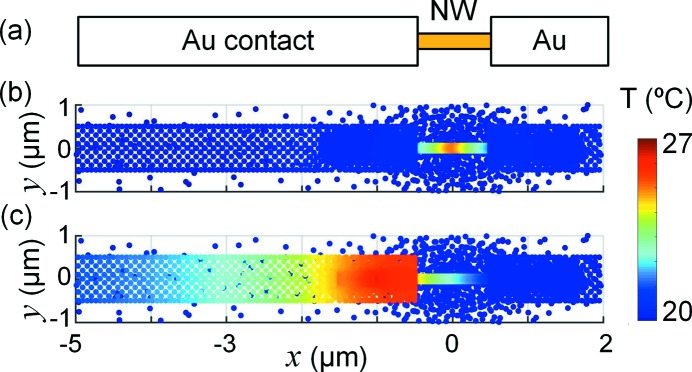
Simulated temperature in a nanowire transistor with 100 nm-thick Au contacts, as viewed along the beam direction, the *z*-axis. The beam properties are the same as in Fig. 3(*a*) ▸. (*a*) Drawing of the sample. (*b*) Temperature when the beam is incident at the centre of the nanowire at *x* = 0. (*c*) Temperature when the beam is incident at the left contact, at *x* = −1 µm. The same colour scale was used in (*b*) and (*c*).

Next, we made steady-state simulations of a set of other sample geometries, with the same beam properties as in the reference case shown in Fig. 3 ▸. First, we studied a nanowire device (Wallentin *et al.*, 2016 ▸), by adding 1 µm-wide and 100 nm-thick Au contacts to a 3 µm-long InP nanowire (heat transfer coefficients to nanowire and substrate *h* = 100 MW m^−2^ K^−1^), shown in Fig. 6(*a*) ▸. Two cases were simulated, with the beam striking either at the centre of the nanowire [*x* = 0, Fig. 6(*b*) ▸] or at the Au contact [*x* = −1 µm, Fig. 6(*c*) ▸]. In the first case, we found that *T*_max_ = 25°C. The Au contacts are efficient heat sinks, which also leads to a strong temperature gradient within the nanowire. When the X-ray instead strikes the Au contact, the temperature is slightly higher, *T*_max_ = 27°C, since the high-*Z* Au contact is an efficient X-ray absorber.

**Figure 7:**
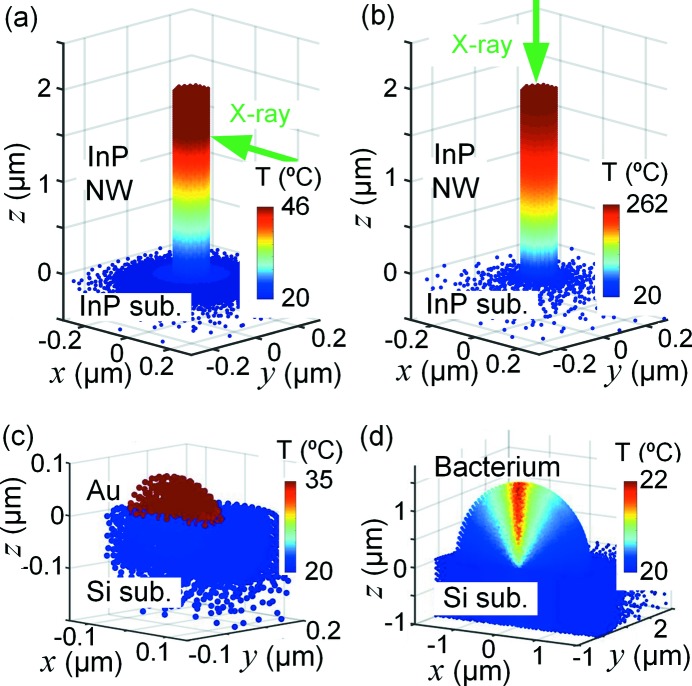
Other sample geometries. (*a*, *b*) InP nanowire on a InP substrate. In (*a*) the beam is parallel to the *x*-axis at *z* = 1.5 µm, and in (*b*) the beam is parallel to the *z*-axis. (*c*) Hemispherical Au nanoparticle, radius 50 nm, on a silicon substrate. (*d*) Hemispherical model bacterium (radius 1.5 µm), on a silicon substrate. In (*c*) and (*d*), only half of the nodes (*y* > 0) are shown for clarity, and the beam is parallel to the *z*-axis.

We also studied an InP nanowire standing as-grown on an InP substrate, a type of sample which has been used by several groups (Diaz *et al.*, 2009 ▸; Robinson & Harder, 2009 ▸; Bussone *et al.*, 2015 ▸; Stankevič *et al.*, 2015 ▸; Dzhigaev *et al.*, 2016 ▸; Thilo *et al.*, 2016 ▸). In this case, there is no heat transfer coefficient between the nanowire and the substrate, but the heat transferring area is small. We simulated both with the beam orthogonal to the nanowire, at *z* = 1.5 µm, and the beam parallel to the nanowire [Figs. 7(*a*) and 7(*b*) ▸]. In the first case, *T*_max_ = 46°C (d*T*_max_ = 26 K), which means that the increase is about three times larger than for the nanowire on the Si_3_N_4_ substrate. For the second case, the maximum temperature is significantly higher, *T*_max_ = 262°C (d*T*_max_ = 242 K), because the absorbing length is about 20 times longer. In both cases, the images show that the nanowire has the same temperature as the substrate at the interface, and that the cooling is limited by the heat conduction within the nanowire.

Another frequently used sample for diffraction experiments is Au nanoparticles (Williams *et al.*, 2003 ▸; Schroer *et al.*, 2008 ▸; Clark *et al.*, 2013 ▸). We simulated a hemispherical Au droplet of radius 50 nm on a silicon substrate, with a heat transfer coefficient of *h* = 100 MW m^−2^ K^−1^. As shown in Fig. 7(*c*) ▸, the temperature in the nanoparticle is fully homogeneous due to the high thermal conductivity of Au. The temperature is higher than in the nanowire, *T*_max_ = 35°C, despite the high heat transfer coefficient, due to the stronger X-ray absorption of Au and the smaller interface area. We note that in such a sample, with high *k* and *h*, the time constants of the cooling are very short and the temporal variation significant.

Finally, we consider a biological sample. One promising application of nanofocused X-rays is imaging of single cells and bacteria (Wilke *et al.*, 2012 ▸; Nam *et al.*, 2013 ▸; Weinhausen *et al.*, 2014 ▸; Pérez-Berná *et al.*, 2016 ▸). The last sample we considered was therefore a model bacterium, with hemispherical shape of radius 1 µm, surrounded by air on a silicon substrate. The heat transfer coefficient at liquid–solid interfaces is generally high, since there is direct contact over the entire interface. The simulations showed a relatively low maximum temperature, *T*_max_ = 22°C, due to the weak absorption. In contrast to the Au nanoparticle, the modest thermal conductivity of the bacterium and the high heat transfer coefficient to the substrate give a strong internal temperature gradient.

## 6. Conclusion   

Our results show that X-ray induced heating can lead to significant sample temperature increases at fluxes that are already available. The time-resolved simulations reveal that immediately after absorption of the X-ray pulse an internal temperature gradient appears. The temperature equilibrates within the nanowire already after a few nanoseconds, due to the high thermal conductivity. Subsequently, the heat dissipates to the substrate, on a slightly slower time scale of tens of nanoseconds. Lower thermal conductivity within the nanowire or a lower heat transfer coefficient to the substrate lead to longer time scales for the cooling, but these still remain far below typical experimental exposure times.

The steady-state simulations demonstrate that the temperature depends on many sample details such as size and thermal conductivity, and that the heat transfer to the substrate is the most important cooling channel. The analytical modelling shows that the nanowire temperature is approximately inversely proportional to the heat transfer coefficient at the nanowire–substrate interface. This parameter varies over many orders of magnitude, and depends on the two solid materials as well as their surface roughness. Air convection dominates the cooling of the nanowire at low values of the heat transfer coefficient or interfacial area. Thermal radiation is very inefficient, which makes samples measured in vacuum particularly vulnerable.

The modelling also demonstrates that significant temperature gradients can appear in samples, both at short time scales and at steady state. These gradients can lead to stress and sample movement, due to local thermal expansion, even if the temperature itself is harmless. The simulations show that both the local and average temperature can depend on the position of the beam on the sample, which means that scanning measurements can lead to significant temperature gradients that vary in time and space.

The development of X-ray nanofocusing concerns both reduction of focus sizes and increased flux. Our simulations indicate that reducing the X-ray focus size is less problematic, since the heat is distributed efficiently at such short length scales. Increasing the flux, however, in general leads to a proportional temperature increase. Thus, the temperature increase is proportional to the absorbed flux, *i.e.* the dose rate, rather than increasing with the integrated dose as is typical for many types of radiation damage. Order-of-magnitude improvements in flux, due to use of pink beam or a diffraction-limited storage ring, can quickly lead to problematic temperatures.

The exact temperatures and dynamics must be determined for each particular case, since nanostructures show a wide variation in geometry and thermal properties. However, modern software and hardware have made finite-element modelling available for non-experts. Some general conclusions regarding promising mitigation strategies can still be drawn. Decreasing the flux is a straightforward remedy, but that also reduces the useful signal and increases measurement time. It seems challenging to reduce the sample temperature by scanning quickly or using short exposure times, since the steady-state temperature is reached within tens of nano­seconds. In our case, a dwell time of 10 ns on a length scale of 100 nm corresponds to a scan speed of 10 m s^−1^, or about 10 µs for a complete scan of a piezo motor with 100 µm range.

Instead, our results suggest that the most promising strategy is to improve the heat transfer to the surrounding. Cryogenically cooled substrates reduce the absolute temperatures, but not necessarily the temperature gradients. One possible approach is to employ a thermal interface material between the object and the substrate, which can increase the heat transfer coefficient by increasing the conducting area. For instance, theoretical modelling has suggested about one order of magnitude lower thermal resistance if water is present at a solid–solid interface (Bahadur *et al.*, 2005 ▸). An extreme approach in this direction would be to immerse the sample in a liquid. Our simulations suggest that the heating induced from next-generation optics and sources should be managed with carefully designed sample environments.

## Supplementary Material

Video 1: Time dependence of the temperature in a nanowire exposed to a single X-ray pulse.. DOI: 10.1107/S1600577517008712/fv5069sup1.avi

Video 2: Time dependence of the temperature in a nanowire exposed to repeated X-ray pulses (period 3 ns).. DOI: 10.1107/S1600577517008712/fv5069sup2.avi
